# New Year’s

**Published:** 1875-12

**Authors:** 


### Editorial.

New Year's.—Once more the year draws to a close, and we await the dawning of another, a *New Year*. What it has in store for us and our readers we cannot say, but most heartily do we wish all a Happy and Prosperous New Year. We have been painfully conscious of the fact that our Journal has at times been quite tardy in its appearance; but with a supply of good material on hand, and the promise of other valuable communications, we promise better for the future. Owing to the press of material we are compelled to present with this issue more than our usual amount of original matter, and as a consequence several Book Reviews and notices have been crowded out. They will appear in our next.

